# DUP1 peptide modified micelle efficiently targeted delivery paclitaxel and enhance mitochondrial apoptosis on PSMA-negative prostate cancer cells

**DOI:** 10.1186/s40064-016-1992-0

**Published:** 2016-03-22

**Authors:** Haining Chen, Fengbo Wu, Jing Li, Xuehua Jiang, Lulu Cai, Xiang Li

**Affiliations:** Department of Gastrointestinal Surgery, Department of Urology, Department of Pharmacy and State Key Laboratory of Biotherapy, West China Hospital, Sichuan University, Chengdu, 610041 China; Department of Pharmacy, Hospital of the University of Electronic Science and Technology of China and Sichuan Provincial People’s Hospital, Chengdu, 610072 China; Department of Clinical Pharmacy and Pharmacy Administration, West China School of Pharmacy, Sichuan University, Chengdu, 610041 China

**Keywords:** Drug delivery, Amphiphilic polymer, Paclitaxel, PSMA-negative, Micelle

## Abstract

Prostate tumor cell targeted peptide fragment conjugated to the nano drug delivery system is a promising strategy for prostate cancer therapy. In this work, an amphiphilic copolymer Chol–PEG–DUP1 (PEG–cholesterol conjugated with DUP1 peptide) has been synthesized and characterized by proton nuclear magnetic resonance spectrum (^1^H NMR). The paclitaxel (PTX) was encapsulated into the Chol–PEG–DUP1 micelles to obtain aqueous formulation with small particle size (within 200 nm) and high drug encapsulating efficiency. The DUP1 modified PTX micelle significantly enhanced the cytotoxicity of paclitaxel to PSMA negative prostate tumor cells (PC-3 cell) as demonstrated by MTT (IC_50_ = 15.8 μg/mL compared to 68.7 μg/mL of free PTX). Flow cytometry analysis and fluorescence images revealed the DUP1 peptide fragments on the surface of micelles increased drug uptake (2.08-fold) by PC-3 cells. Flow cytometry and immunoblotting analysis showed the DUP1 modified PTX micelle enhanced the mitochondrial apoptosis-inducing capacity of PTX to PC-3 cells. In conclusion, Chol–PEG–DUP1 modified micelle was a reasonable, facile, and economic drug delivery system to target the PSMA-negative prostate cancer.

## Background

Nowadays, prostate cancer is one of the main lethal cause from cancer patients worldwide, and more than one-third of newly diagnosed male cancer in Europe and USA was prostate cancer (Crawford 2003; Parkin et al. 2001). Despite the hormone therapy was effective in early stage, many patients with metastatic potential eventually progress to an androgen-resistant state (Sandblom and Varenhorst 2001; Zitzmann et al. 2005). Although there were several treatment methods applied in the clinic, but none showed a survival benefit in hormone independent prostate cancer patients (Sternberg 2003).

Prostate-specific membrane antigen (PSMA) as a member of trans-membrane folate hydrolase family, which could enhanced the expression level in prostate cancer tissue other than benign or neoplastic epithelial prostate cells (Bostwick et al. 1998; Ross et al. 2003). A weak extra prostatic expression of PSMA has been reported in some other tissues (Renneberg et al. 1999; Silver et al. 1997) Therefore, the Hu591 monoclonal antibody(mAb) targeting the PSMA extracellular domain, has been applied to the prostate cancer therapy (Liu et al. 1997; Nanus et al. 2003). An ^111^In-labeled monoclonal antibody (Capromab pendetide, ProstaScint) could targeted to PSMA and used for imaging lymph node metastases. Recently, Zitzmann et al., have reported a novel peptide DUP1 with specificity for PSMA-negative prostate tumor cell lines, such as DU-145 and PC-3, which was identified by phage display techniques.

Peptide modified polymeric micelles have been investigated extensively, and these works are quite extraordinary, impressive, and laid a solid foundation for our study. In recent years, Torchilin et al., have reported that the monoclonal antibody (mAb)-modified PEG-PE micelles could recognize and bind to numerous tumor cells but not normal cells in vitro (Torchilin et al. 2003). Several researchers have reported that the synergetically therapeutic efficacy of chemotherapy by polymer–peptide or drug–peptide conjugates, which leads to amplifying apoptosis induction activity in the tumor or enhancing tumor targeting (Liu et al. 2008; Dharap et al. 2005). In our previously studies, we have reported the fibroblast growth factor (FGF) fragment peptide modified micelles could significantly enhanced the cytotoxicity of paclitaxel for murine lewis lung cancer (LLC) cells, which were further confirmed by some subsequent experiments in vitro (Cai et al. 2011). These antibodies or peptides conjugated micelles could enhanced the specific uptake of drugs and/or genes by targeted cells actively. To our knowledge, nanotechnology is widely applied to prepare a novel nano-formulation of hydrophobic drugs for enhancing cancer therapy efficiency (Gong et al. 2013). By encapsulating into nanoscale vectors, hydrophobic drugs could form stably disperse morphology in water (Wagner et al. 2006; Jain and Stylianopoulos 2010; Li et al. 2012). Moreover, amphiphilic polymeric micelles have widely applied in drug delivery system (DDS) for anti-tumor agents, in which the hydrophilic shell helps the escape from the clearance by RES (reticuloendothelial system) and the hydrophobic core wraps drug via hydrophobic interactions or hydrogen bonds (Gong et al. 2010, 2011; He et al. 2012; Li et al. 2012, 2013; Wu et al. 2012; Ma et al. 2013; Zhang et al. 2014; Zeng et al. 2014). Furthermore, their nano-size will improve the anti-tumor effects through the enhanced permeability and retention (EPR) effect (Iyer et al. 2006; Muggia 1999; He et al. 2013).

**Fig. 1:**
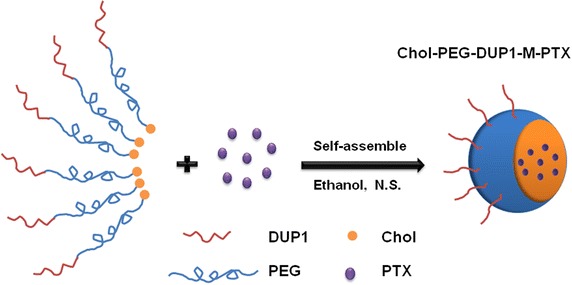
Schematic representation of Chol–PEG–DUP1–M-PTX

In this work, DUP1 peptide was obtained by solid phase peptide synthesis, and DUP1 modified polymeric micelles were prepared using a solid dispersion method (Fig. 1). Cytotoxicity, apoptosis, cellular uptake, and drug release behavior of paclitaxel (PTX) loaded Chol–PEG–DUP1 micelles (Chol–PEG–DUP1–M-PTX) were studied in detail. Hence, this novel tumor targeting DDS may have several functions for prostate tumor therapy: first of all, the Chol–PEG–DUP1 micelles could provide prolonged drug effects because of their sustained release characteristic, and protect the encapsulated agent from enzymatic degradation; secondly, the nanoscale size of the micelles would improve the targeting efficiency and therapeutic activity of small molecular antitumor drug via EPR effect; finally, the DUP1 fragment could specifically target to the PSMA-negative tumor cells, resulting in special uptake of antitumor drugs.

## Methods

### Materials and cell lines

Cholesterol (Chol) monomethoxy poly(ethylene glycol) (MW 2000, mPEG2000) and poly(ethylene glycol) (MW 2000, PEG2000), was obtained from BoAo Biological Technology (Shanghai, China). The 3-[4, 5-dimethylthiazol-2-yl]-2, 5-diphenltetrazolium bromide (MTT), 1-(3-dimethylaminopropyl)-3-ethylcarbodiimide hydrochloride (EDCI), and 4-dimethylaminopyridine (DMAP) 1, 8-Diazabicyclo(5.4.0)undec-7-ene (DBU), Coumarin-6, fmoc–l-phenylalanine (fmoc–phe), and succinic anhydride (suc) were Obtained from Sigma-Aldrich (St. Louis, MO, USA). 3-Maleimidopropionic acid N-succinimidyl ester (BMPS) was purchased from Jiaxing Biomatix Co. Ltd. (Jiaxing, China). Soya phosphatidylcholine (SPC) was from Lucas Meyer (Hamburg, Germany). Paclitaxel (PTX) was purchased from Energy Chem. Co. Ltd. (Shanghai, China).

The DUP-1 peptide (CFRPNRAQDYNTN) was synthesised by standard solid-phase peptide synthesis method using Fmoc chemistry. The DUP1 peptide was purified by preperative high-pressure liquid chromatography on a Novasep LC50, C_18_-ODS-5 μm, 250 × 50 mm column (Novasep, Pompey, France) using water, acetonitrile and methanol as eluent solvent. All other solvents and reagents were of chemical grade and used without other purification. The ultrapure water was prepared from Milli-Q water system without specification.

PC-3 cell was purchased from the American Type Culture Collection (ATCC, Rockville, MD, USA). PC-3 cells grew in Roswell Park Memorial Institute 1640 medium (RPMI 1640, Gibco, Grand Island, NY, USA) supplemented with 10 % fetal bovine serum (FBS, Gibco, USA). All cells were maintained at 37 °C in humidified incubator containing 5 % CO_2_.

### Synthesis of cholesterol–poly(ethylene glycol)–DUP1 peptides copolymers

**Scheme 1:**
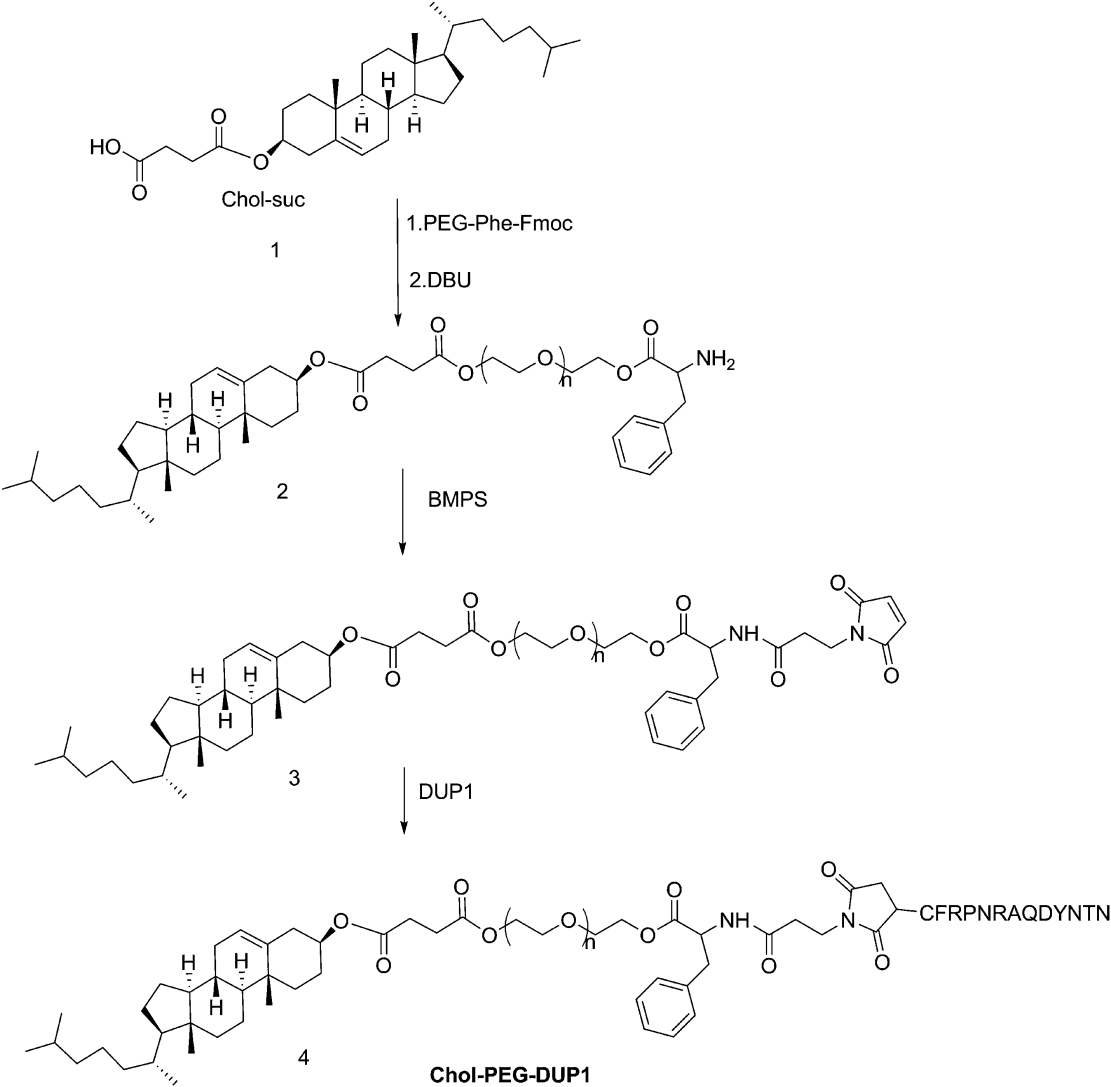
Synthetic route of Chol–PEG–DUP1 copolymers

The synthesis process of cholesterol–poly(ethylene glycol)–DUP1 copolymers (Chol–PEG–DUP1) was followed our previous report; the synthetic route was showed in Scheme 1.

In general, the DMSO solution of Chol–PEG–Phe-NH_2_ (2.0 g, 0.76 mmol), and BMPS (0.24 g, 0.92 mmol) was stirred 24 h at room temperature to give a 3-maleimidopropionic acid modification on the PEG chain. Then the DUP-1 peptide (CFRPNRAQDYNTN, 1.51 g, 0.92 mmol) was added and continuous stirred 12–24 h. After the reaction was completed, the solution was dialyzed to remove un-reacted peptides (membrane tubing, molecular weight cut off = 1.0 KD). The dialyzed product was lyophilized and the final products Chol–PEG–DUP1 (1.42 g, 47.1 %) was obtained.

### Preparation and characterization of Chol–PEG–DUP1–M-PTX

The paclitaxel loaded Chol–PEG–DUP1 micelles (M-PTX) were prepared as following: PTX and copolymer (1:19, w/w) were dissolved in ethanol, and the solution was evaporated in rotary evaporator for 20 min to obtain the co-evaporation. Then, normal saline (NS) was added, and co-evaporation was dissolved to self-assemble into Chol–PEG–DUP1–M-PTX. The obtained Chol–PEG–DUP1–M-PTX was first filtered using a 0.22 μm Millex-LG filter (Millipore Co., Waltham, MA, USA), and then lyophilized into powder form before use.

The Malvern Nano-ZS 90 laser particle size analyzer (Malvern, Worcestershire, UK.) was utilized to the determination of particle size distribution and zeta potential of Chol–PEG–DUP1–M-PTX. All the results were tested in three different samples, and data were expressed as the mean ± standard deviation (SD). The morphological characteristic of Chol–PEG–DUP1–M-PTX was further detected by transmission electron microscope (TEM, H-6009IV, Hitachi, Tokyo, Japan). The Chol–PEG–DUP1–M-PTX sample was negatively staining by phosphotungstic acid before TEM test.

The high performance liquid chromatography (HPLC, Waters Alliance 2695, Milford, MA, USA) instrument was used to determine the drug loading (DL) and encapsulation efficiency (EE) of Chol–PEG–DUP1–M-PTX with a ultra-violet (UV) detector, and chromatographic separations were performed on a reversed phase C_18_ column (4.6 × 150 mm, 5 μm, Inertsil/WondaSil, Japan). The DL and EE of Chol–PEG–DUP1–M-PTX were calculated according to the following equations:

1 $${\text{DL}} = {\text{Drug}}/\left( {{\text{Drug}} + {\text{Polymer}}} \right) \times 100\,\%$$

2 $${\text{EE}} = {\text{Drug in micelles}}/{\text{drug in feed}} \times 100\,\%$$

### In vitro cytotoxicity

To investigate cytotoxicity of Chol–PEG–DUP1–M-PTX and free PTX, MTT assays were preformed on PC-3 cells. PC-3 cells cultured in 96-well plates were treated with a series of Chol–PEG–DUP1–M-PTX or free PTX for 48 h, respectively. The mean percentage of cell survival relative to that of control cells was determined from data of three individual experiments, and all the data were expressed as mean ± SD.

### Apoptosis assay

Apoptosis induction assay of Chol–PEG–DUP1–M-PTX and free PTX were studied on PC-3 cells. PC-3 cells were plated in 6-well plates and grown for 24 h. The cells were exposed to media containing 20 ng/mL of Chol–PEG–DUP1–M-PTX and free PTX for 48 h, respectively. Then, the cells were fixed with pre-chilled 70 % ethanol for 30 min and stained with 0.5 mL of PI (5 μg/mL in PBS) for 10 min. Apoptotic cells were observed under fluorescence microscopy (TE2000-U, Nikon, Tokyo, Japan), which demonstrated cytoplasmic and nuclear shrinkage and chromatin condensation.

Furthermore, flow cytometric (FCM) assay was used to confirm the apoptotic induction effect of Chol–PEG–DUP1–M-PTX. Apoptosis of PC-3 cells treated with PTX -M, free PTX, or blank micelles was determined using FITC-conjugated AnnexinV/PI (BD PharMingen, San Diego, CA, USA) staining by FCM (BD FACS Calibur, BD, San Jose, CA, USA). Both early apoptotic (Annexin V^+^/PI^−^) and late apoptotic (Annexin V^+^/PI^+^) cells were included in cell apoptosis determinations.

### In vitro drug release

The release profiles of PTX from DUP1-modified micelles or free PTX were investigated by the dialysis method. Briefly, 1 mL of free PTX solution or drug loaded micelles were placed into dialysis bags (molecular weight cut off = 3500), then incubated at 37 °C with gentle shaking (100 rpm) in 50 mL of phosphate buffered solution (PBS) (PH 7.4 or PH 5.5, 0.01 M) containing Tween80 (0.5 wt%). After given time intervals, dialysis medium was withdrawn and replaced with the same volume of fresh buffer. The cumulative amount of released PTX were analyzed and quantified by HPLC. All the results were the mean value of three test runs and all data were shown as the mean ± SD.

### Cellular uptake of micelles by flow cytometry analysis

In general, the PC-3 cells suspension (6 × 10^4^ cells/well in 1.5 mL) were incubated at 37 °C for 24 h in six-well plates (Corning, NY, USA). Then conventional micelles or DUP1-modified micelles loading the same amount of coumarine-6 (the final concentrations were about 40 ng/mL) were added into each well, respectively. Then the six-well plates were further incubated at 37 °C for 1 h, the culture medium was discarded, the plates were digested with trypsin and the re-suspended cells were washed with cold PBS twice. Finally, each sample was examined by a flow cytometer (EPICS Elite ESP, Beckman Coulter, Brea, CA, USA). The fluoresces of intracellular coumarin-6 was excited at 488 nm with an argon laser, and the emission fluorescence was detected at 525 nm. Files were collected of 10,000 gated events.

### Immuno-blotting analysis

The cellular total proteins were extracted using RIPA buffer (SolarBio, Beijing, China) containing 1 % (*v*/*v*) PMSF (SolarBio), 0.3 % (*v*/*v*) protease inhibitor (Sigma, St. Louis, MO, USA) and 0.1 % (*v*/*v*) phosphorylated proteinase inhibitor (Sigma). Celluar lysates were centrifuged at 13,000 rpm at 4 °C for 10 min, the supernatant was collected. The protein concentration was determined using BCA protein assay kit (Pierce, Waltham, MA, USA). The total protein was separated on SDS-PAGE gel and transferred onto a PVDF membrane. Non-specific interactions were blocked using skimmed milk for 2 h at room temperature. The PVDF membranes were incubated with the primary antibodies overnight at 4 °C. After washed several times, the PVDF membranes were incubated in HRP-conjugated goat anti-rabbit and anti-mouse IgG or HRP-conjugated mouse anti-goat IgG (Abmart, Shanghai, China, all at a 1:5000 dilution) for 4–8 h at room temperature. The target proteins were visualized using enhanced chemiluminescence (Millipore, Billerica, MA, USA) according to the manufacturer’s recommendations.

## Results and discussion

### Synthesis and characterization of paclitaxel loaded micelles with or without DUP1 modification

**Fig. 2:**
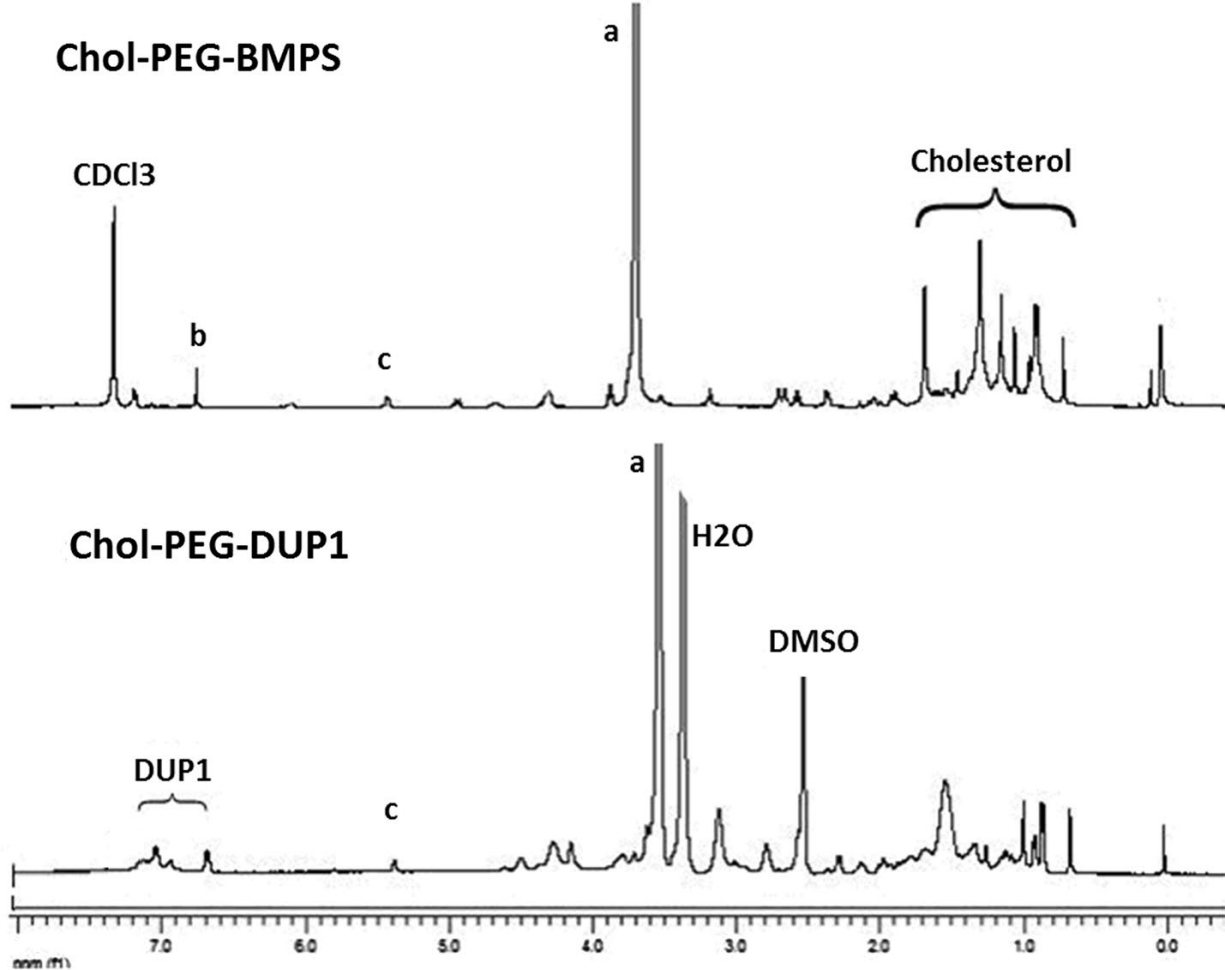
The ^1^H nuclear magnetic resonance spectrum (^1^H NMR) spectrum of Chol–PEG–BMPS and Chol–PEG–DUP1 copolymers

The synthesis of Chol–PEG–DUP1 was according to our previous reports with some modifications (He et al. 2012; Zeng et al. 2014). Figure 2 represents the ^1^H NMR spectrum of Chol–PEG–BMPS (PEG–cholesterol conjugate of (N-[β-maleimidopropyloxy] succinate) and Chol–PEG–DUP1, demonstrated the successful synthesis of Chol–PEG–DUP1. As shown in Fig. 2, the multiplet peaks at δ 3.47–3.76 were attributed to the methylene group of repeating ethylene glycol units in PEG, and the six- and three position protons in cholesterol were found as single peak at 5.36 and 4.59 ppm, respectively. The multiplet peaks around 4.24 ppm were assigned to the PEG methylene protons near the succinyl group, and the multiplet peaks at 2.70 were attributed to the methylene proton of succinyl group. All of the other multiple signals at 0.68–2.05 were the signals the protons in cholesterol. These results further showed that the conjugate had been successfully prepared.

**Fig. 3:**
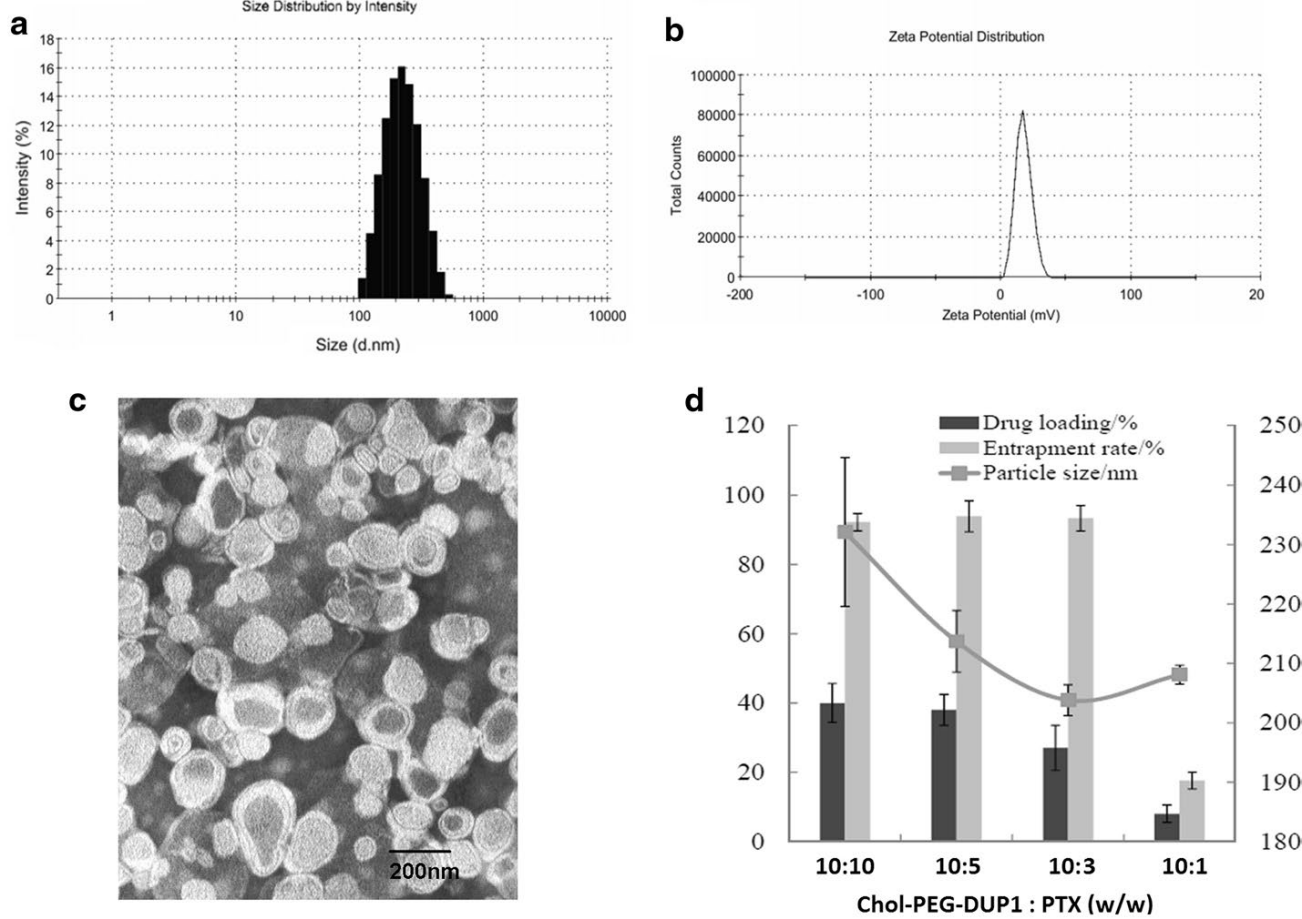
Characterization of Chol–PEG–DUP1–M-PTX. **a** Particle size of Chol–PEG–DUP1–M-PTX; **b** zeta potential of Chol–PEG–DUP1–M-PTX; **c** TEM image of Chol–PEG–DUP1–M-PTX; **d** the effect of polymer/drug ratio on the encapsulation efficiency (EE), drug loading (DL) and size of Chol–PEG–DUP1–M-PTX [mean ± SD (standard derivations), *n* = 3]

In our study, Chol–PEG–DUP1–M-PTX was prepared by a one-step solid dispersion method, the mean size of paclitaxel loaded micelles were fluctuated from 202 to 230 nm, with the different drug/polymer (*w*/*w*) ratio. The optimized polymer/drug ratio was 10:3, the optimized DL and EE were 24.9 ± 0.6 % and 96.40 ± 1.8 %, respectively. Furthermore, particle size, polydisperse index (PDI), and zeta potential of obtained PTX loaded Chol–PEG–DUP1 micelles were 202.5 ± 1.0 nm, 0.218 ± 0.032, and 12.6 ± 3.4 mV, respectively (Fig. 3a, b). Transmission electron microscopy (TEM) image of Chol–PEG–DUP1–M-PTX was exhibited in Fig. 3c, and it indicated that the Chol–PEG–DUP1–M-PTX was spherical in shape with a diameter of about 200 nm. The results of particle size analysis and microstructure of PTX observed by TEM suggested that a homogenous and stable solution of Chol–PEG–DUP1–M-PTX could be achieved by encapsulating PTX into polymeric micelles.

### In vitro drug release profile

**Fig. 4:**
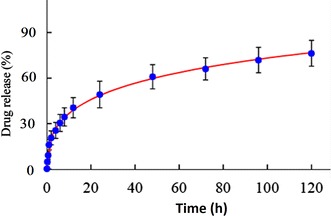
Time course of paclitaxel releasing from Chol–PEG–DUP1 micelle at 37 °C and pH 7.4. Released paclitaxel was separated by dialysis and quantified using high performance liquid chromatography (HPLC, *error bars* correspond to 95 % confidence intervals)

The in vitro release profile of paclitaxel from DUP1 peptide modified micelles (Chol–PEG–DUP1–M-PTX) was studied at 37 °C and pH 7.4. Data suggest that paclitaxel can be well encapsulated in Chol–PEG–DUP1 micelles, and released in an extended period. As shown in Fig. 4, approximately 54 % of total drugs released after 24 h, followed by release of 78 % in 120 h. This cumulative drug release suggests potential applicability of these micelles as promising DDS that could result in a content pharmacokinetic profile in vivo and reduced exposure of healthy tissues.

### Cell uptake and binding study

**Fig. 5:**
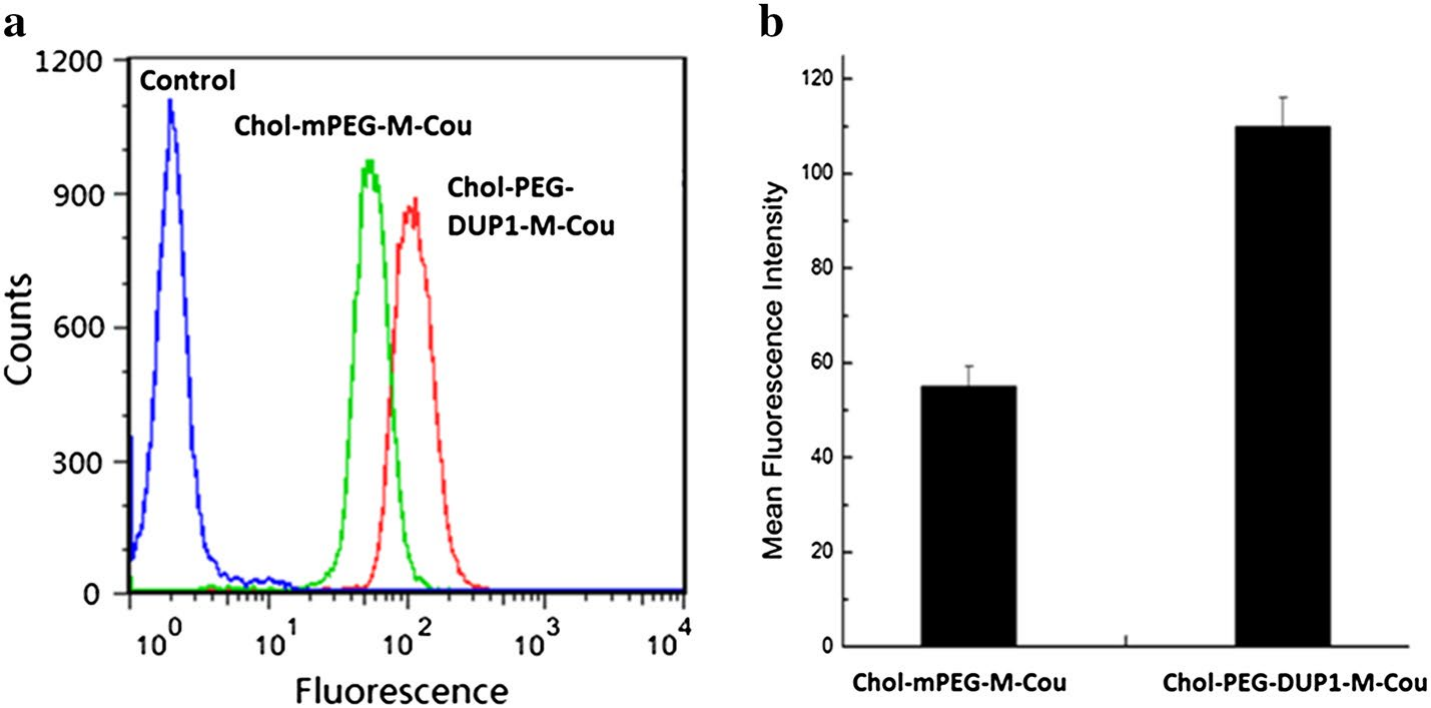
Different cellular uptake of coumarine-6 loading micelles with or without DUP1 by PC-3 tumor cells. **a** Intracellular coumarine-6 fluorescence intensities in PC-3 cells after incubation with Chol–PEG–DUP1–M-Cou (coumarine-6 loaded Chol–PEG–DUP1 micelles), Chol–mPEG–M-Cou (coumarine-6 loaded Chol–mPEG micelles), or empty Chol–PEG–DUP1 micelles (Control); **b** quantification of the mean fluorescence intensities of intracellular coumarine-6 from Chol–PEG–DUP1–M-Cou or Chol–mPEG–M-Cou, respectively

**Fig. 6:**
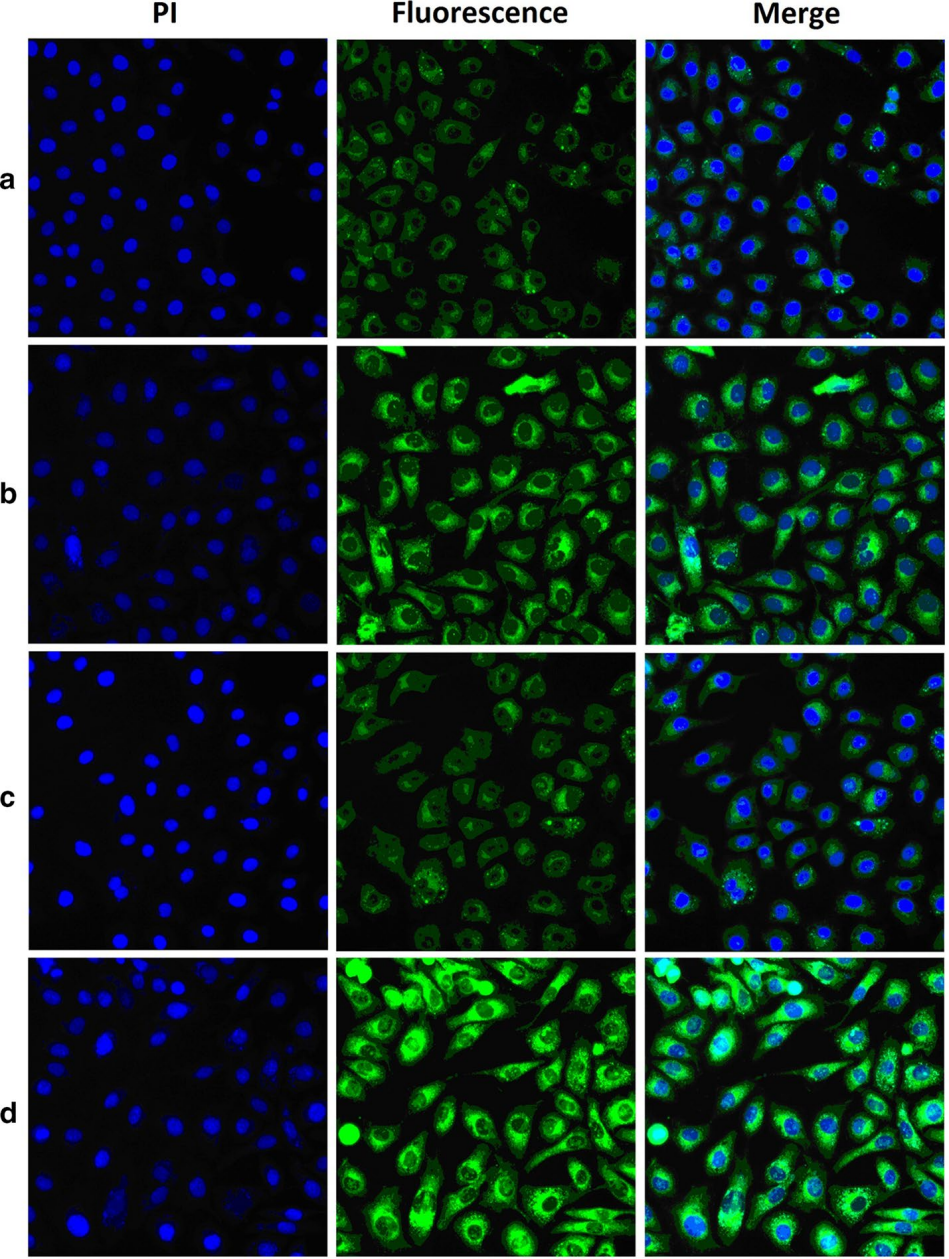
The fluorescence microscopy images (×40) of PC-3 tumor cells after incubation with different coumarine-6 loaded micelles: Control (**a**), the Chol–mPEG–M-Cou (**b**), free DUP1 + Chol–PEG–DUP1–M-Cou (**c**) and the Chol–PEG–DUP1–M-Cou (**d**)

To evaluate whether DUP1 peptide fragments could increase drug uptake by PSMA negative prostate tumor cells, PC-3 cells were treated with micelles encapsulated with coumarine-6. After incubation for a specific time, the cells were washed, then identified using fluorescence microscopy and collected for analysis of coumarine-derived fluorescence by flow cytometry. The flow cytometry data (Fig. 5a) shows the distribution of intensity in PC-3 cells treated with Chol–PEG–DUP1–M-Cou, Chol–mPEG–M-Cou, or empty Chol–PEG2000–DUP1 micelles (Control). Mean fluorescence intensity of coumarine-6 uptake by cells treated with Chol–PEG–DUP1–M-Cou was increased by 2.08-fold (mean fluorescence intensity = 54.7 vs. 113.8, *p* < 0.05) compared with that treated with Chol–mPEG–M-Cou (Fig. 5). These results indicate that the DUP1 peptide fragments on the surface of micelles indeed enhanced the uptake of the drug (coumarine-6) in tumor cells. Which were further confirmed by fluorescence microscopy images (Fig. 6). Figure 6 is supported Chol–PEG–DUP1–M-Cou could effectively entry PC-3 prostate tumor cells, while less coumarine-6 was detected in cells that incubated with Chol–mPEG–M-Cou. When the PC-3 tumor cells were pre-incubated by free DUP1 peptides, the cellular uptake of Chol–PEG–DUP1–M-Cou was obviously decreased, this further confirms the selectivity of Chol–PEG–DUP1 micelles to tumor cells were came from the DUP1 peptide fragments.

### In vitro cytotoxicity evaluation

**Fig. 7:**
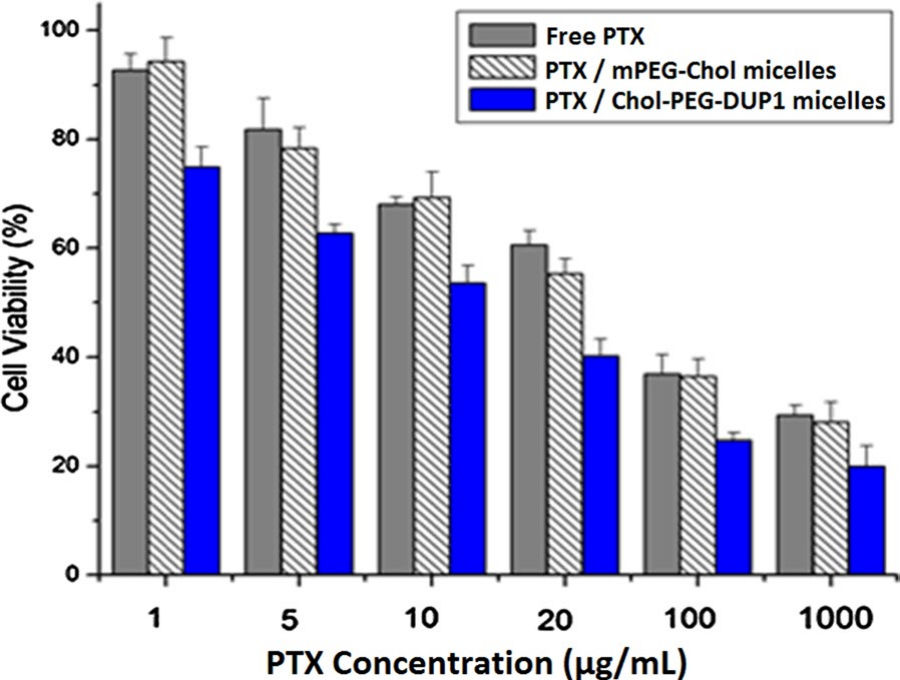
The in vitro cytotoxicity of the free PTX, Chol–mPEG–M-PTX, and Chol–PEG–DUP1–M-PTX on PC-3 cell lines (48 h). The percentage of viable cells was quantified using the methylthiazoletetrazolium method. Mean values and 95 % confidence intervals derived from three independent experiments are shown, *p < 0.05

In vitro cytotoxicity of Chol–PEG–DUP1–M-PTX and free PTX was determined by cell viability assay on PC-3 cells, and the results were shown in Fig. 7. With increase of PTX concentration, cell viabilities were decreased accordingly in all groups, but there was no significant difference between Chol–mPEG–M-PTX and free PTX groups. After 48 h incubation with the free PTX, Chol–mPEG–M-PTX or Chol–PEG–DUP1–M-PTX, cytotoxicity was measured following the absorbance of the degraded MTT at 570 nm. As shown in Fig. 7, Chol–PEG–DUP1–M-PTX showed significantly higher cytotoxicity than free paclitaxel or Chol–mPEG–M-PTX. The mean concentrations of paclitaxel that caused 50 % cell inhibition (IC_50_) of Chol–PEG–DUP1–M-PTX was decreased to 15.83 μg/mL compared with 68.67 μg/mL of free paclitaxel and 65.76 μg/mL of Chol–mPEG–M-PTX, respectively.

### In vitro apoptosis induction effect

**Fig. 8:**
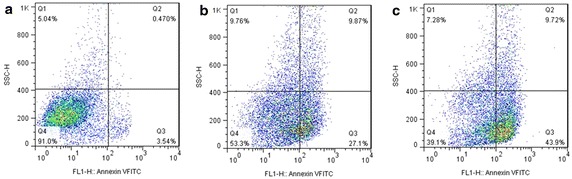
Flow cytometric analysis of PC-3 cells stained with Annexin V-FITC/PI after treatment with control group (**a**), 10 μM of free PTX (**b**) or Chol–PEG–DUP1–M-PTX (**c**)

In vitro apoptosis induction effect of Chol–PEG–DUP1–M-PTX was investigated using FCM assay of Annexin V/PI staining was employed to investigate the apoptosis induction effect of Chol–PEG–DUP1–M-PTX, and both early apoptosis (Annexin V^+^/PI^−^) and late apoptosis (Annexin V^+^/PI^+^) cells were included. According to Fig. 8, the percentage of apoptotic cells in Chol–PEG–DUP1–M-PTX group was 52.62 ± 7.12 %, which was significantly higher than that in free PTX (36.97 ± 5.75 %, *p* < 0.05), and NS (4.01 ± 0.74 %, *p* < 0.05) groups. There was no significant difference in late apoptosis between the Chol–PEG–DUP1–M-PTX (9.72 ± 2.15 %) and free PTX (9.87 ± 2.59 %, *p* < 0.05) groups, but early apoptotic cells in the Chol–PEG–DUP1–M-PTX group (43.9 ± 5.09 %) were more than that in free PTX group (27.1 ± 3.64 %, *p* < 0.05). The results of morphological observation and FCM assay suggested that, compared with free PTX, Chol–PEG–DUP1–M-PTX induced more apoptotic cells.

### Immunoblotting analysis

**Fig. 9:**
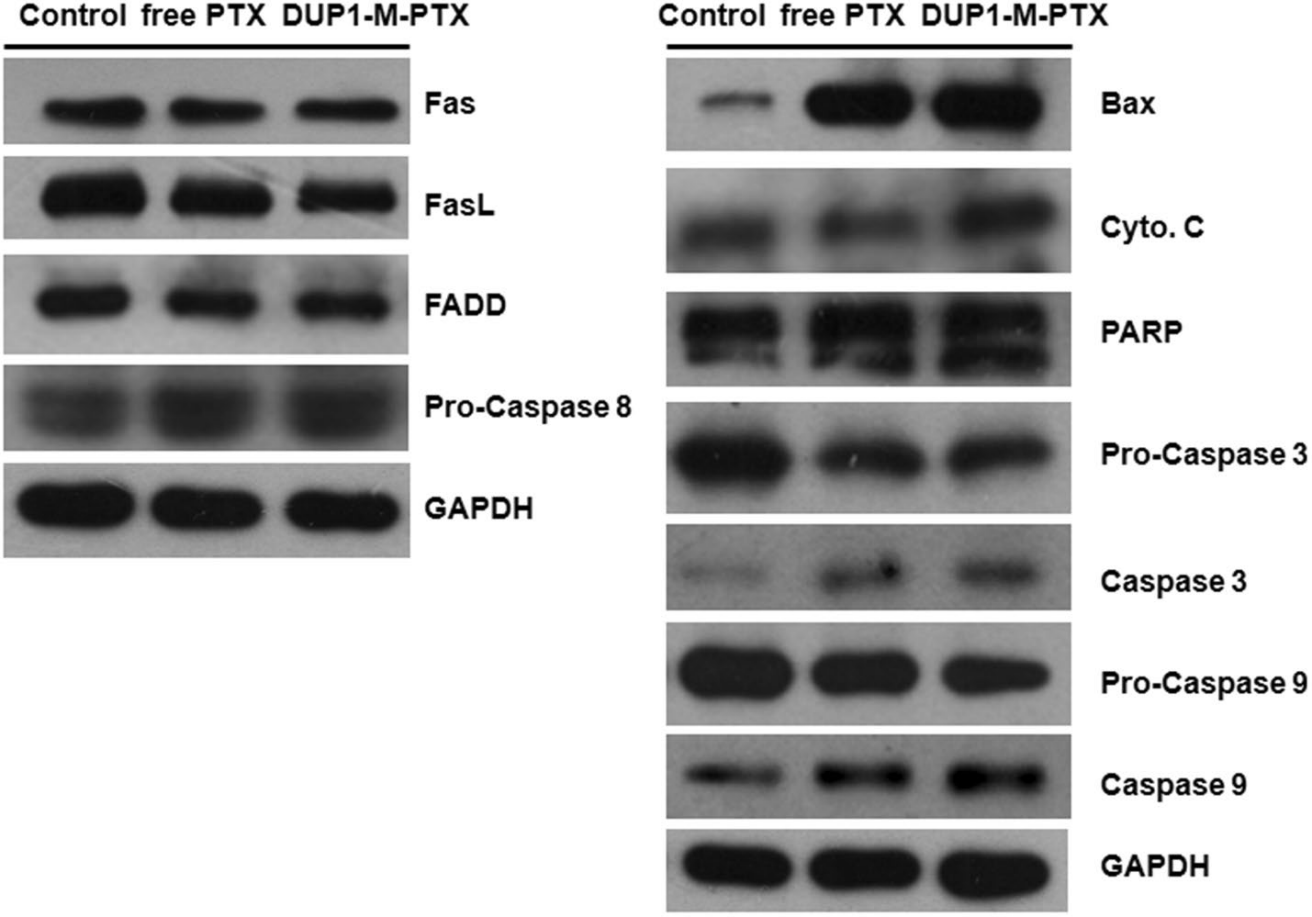
PTX and Chol–PEG–DUP1–M-PTX induces apoptosis via mitochondrial pathways in human prostate cancer PC-3 cells, as detected by Western blot analysis, glyceraldehyde phosphate dehydrogenase (GAPDH) served as a loading control

To validate the expression of apoptosis related proteins in Chol–PEG–DUP1–M-PTX and free PTX treated PC-3 cells, immunoblotting assays were performed using examples of the death receptor pathway and mitochondrial pathway related to apoptotic proteins. Generally, apoptotic cell death is triggered by either the intrinsic pathway involving mitochondria or by the extrinsic pathway involving death receptors. The death receptor pathway can be initiated by stimulation of members of the death receptor family such as Fas, tumor necrosis factor related apoptosis-inducing ligand (TRAIL), and TNF-α. Initial mechanistic studies with Chol–PEG–DUP1–M-PTX and free PTX showed that the drug and drug loaded micelle only slightly increased levels of FasL, Fas-Associated protein with Death Domain (FADD), and cleaved caspase-8 in PC-3 cells (Fig. 9). This suggests that the apoptotic procedure barely involves the death receptor pathway. At the same time, Chol–PEG–DUP1–M-PTX and free PTX increased the amounts of Bax and cytosolic cytochrome c (Fig. 9), indicating that at least part of PTX and DUP1-M-PTX induced apoptosis occurs via mitochondrial pathway. Since the release of cytochrome c from mitochondria can activate the proapoptotic caspase cascade, we investigated whether caspases 3 and 9 are involved. Treating PC-3 cells with PTX and drug loaded micelle increased levels of the cleaved forms of caspases 3 and 9 (Fig. 9). These results provided additional evidence that PTX induced apoptosis mainly occurred in the mitochondrial pathway, and the DUP1 modified micelle could enhance the apoptosis-inducing capacity of PTX to PC-3 prostate cancer cells.

## Conclusions

In conclusion, targeting peptide modified PEG–Chol polymeric micelles, the surfaces of which are conjugated with DUP1 peptide, have been prepared for PSMA negative PCa targeted drug delivery. The reported Chol–PEG–DUP1–M-PTX bearing both small particle size and high encapsulating efficiency. In vitro experiments suggested that Chol–PEG–DUP1–M-PTX had prior cytotoxicity than free drug in the cell proliferation MTT assays, and could inducing more apoptosis. Cellular flow cytometry results and fluorescence spectroscopy images suggest the surface DUP1 modification of the micelles promote the selective uptake by PSMA negative PC-3 cells. Although further in vivo antitumor investigation of Chol–PEG–DUP1–M-PTX is required, the results of our current study represent a meaningful explore in advancing the use of DUP1 peptide modified micelles as a potent strategy to treat PSMA negative prostate cancer. Thus, Chol–PEG–DUP1–M-PTX may serve as a promising candidate for prostate cancer therapy.
